# ﻿*Gastrochilusobovatifolius* (Orchidaceae, Aeridinae), a new species from the Daba Mountains of Chongqing, China

**DOI:** 10.3897/phytokeys.252.133501

**Published:** 2025-02-07

**Authors:** Chi Xiong, Xiao-Ying Fu, Ke Tan, Ya Huang, Hong-Jing Zhang, Yi-Chen Wang, Yu-Bing Yang, Si-Rong Yi

**Affiliations:** 1 Guangxi Key Laboratory of Plant Conservation and Restoration Ecology in Karst Terrain, Guangxi Institute of Botany, Guangxi Zhuang Autonomous Region and Chinese Academy of Sciences, Guilin, 541006, Guangxi, China Guangxi Key Laboratory of Plant Conservation and Restoration Ecology in Karst Terrain, Guangxi Institute of Botany, Guangxi Zhuang Autonomous Region and Chinese Academy of Sciences Guilin China; 2 College of Life Sciences, Guangxi Normal University, Guilin 541006, Guangxi, China Guangxi Normal University Guilin China; 3 Chongqing Key Laboratory of Development and Utilization of Genuine Medicinal Materials in Three Gorges Reservoir Area, Chongqing Three Gorges Medical College, Wanzhou, Chongqing, 404120, China Chongqing Key Laboratory of Development and Utilization of Genuine Medicinal Materials in Three Gorges Reservoir Area, Chongqing Three Gorges Medical College Chongqing China; 4 Hangzhou Botanical Garden, Hangzhou, 310013, China Hangzhou Botanical Garden Hangzhou China; 5 Center for Biodiversity Conservation and Utilization, Key Laboratory of Eco-Environment in the Three Gorges Reservoir Region, Ministry of Education, School of Life Sciences, Southwest University, 400715, Beibei, Chongqing, China Southwest University Chongqing China

**Keywords:** Epidendroideae, Flora of Chongqing, *G.sect.Microphylli*, phylogeny, Qinling-Daba Mountains, taxonomy

## Abstract

*Gastrochilusobovatifolius*, a new species discovered in the Dabashan of northeast Chongqing (China), is described and illustrated. The novelty morphologically resembles *G.affinis*, *G.balangshanensis* and *G.heminii* in having a glabrous and smooth epichile. but can be distinguished by the unique obovate shape of the leaves, smaller epichile, and flower color. The results of molecular phylogenetic analysis, based on nuclear ribosome internal transcribed spacer (nrITS) and four chloroplast DNA fragments (*mat*K, *psb*A–*trn*H, *psb*M–*trn*D, and *trn*L–F) of 55 *Gastrochilus* species, support the inclusion of *G.obovatifolius* in G.sect.Microphylli, being most closely related to *G.balangshanensis* from Sichuan. The new species is a trunk epiphyte in evergreen broad-leaved forest.

## ﻿Introduction

The orchid genus *Gastrochilus* D.Don (Don 1825: 32) part of the mostly Asian vandoid subtribe Aeridinae, comprises approximately 80 species distributed in tropical and subtropical Asia, from India and Sri Lanka to East Asia and south to Indonesia (Tsi 1996, 1999; Liu et al. 2019; Zhang et al. 2022; Ya et al. 2023; POWO 2024; Zhang et al. 2024a, b). Among these, around 60 species are found in China (including approximately 40 endemic), where new species continue to be regularly described (Chen et al. 2009; Liu 2017; Liu et al. 2019, 2023; Li et al. 2022; Ya et al. 2023; Zhang et al. 2024a, b; Zhou et al. 2024). A recent systematic study has classified *Gastrochilus* into six sections: Gastrochilussect.Gastrochilus, G.sect.Pseudodistichi Jun Y. Zhang & H. He, G.sect.Brachycaules Q. Liu & J.Y. Gao ex Jun Y. Zhang & H. He, G.sect.Acinacifolii Q. Liu & J.Y. Gao ex Jun Y. Zhang & H. He, G.sect.Microphylli (Benth. & Hook. f.) Seidenf., and G.sect.Caespitosi Z. H. Tsi (Zhang et al. 2024a). These sections can be delimited by leaf shape and length, type of epichile hairs and margin, and by whether the epichile surface is smooth or not (Zhang et al. 2024a). Recently, the Mountains of Southwest China have emerged as a fertile ground for new species discovery in G.sect.Microphylli, namely the Hengduan Mountains (Liao et al. 2022, Ya et al. 2023, Zhang et al. 2024b), but the Qinling-Daba Mountains, to the east, have received less attention.

The Qinling-Daba Mountains (QDM) constitute a comprehensive geographical and geomorphic entity, serving as a natural demarcation between northern and southern China. These mountains function as an important corridor between the Hengduan Mountains and the eastern plains, separating the warm temperate zone from the subtropical zone (Zhu 1958, Zhang 2019). This region is acknowledged as one of China’s foremost biodiversity hotspots, hosting a total of 9,491 species of seed plants, accounting for 39% of the overall seed plant diversity present in China (Zhang et al. 2022a). Moreover, the area is home to 3,585 endemic species, which represent 37% of the vascular plant species found within this region (Zhang et al. 2022b). In March 2024, during a plant survey in Chengkou County, Northeast Chongqing, at the southern foothills of the QDM, we encountered an unknown species of *Gastrochilus* with distichous, alternate, and obovate leaves, and small flowers with dark purple stripes. The smooth and glabrous epichile pointed to its inclusion in G.sect.Microphylli (Seidenfaden 1988). Some living plants were put into cultivation at Guilin Botanical Garden for further study. Standard herbarium taxonomy, including a comprehensive literature review on the genus (Chen et al. 2009; Liu 2017; Lee et al. 2019; Liu et al. 2019; Rao et al. 2019; Wu et al. 2019; Ormerod and Kumar 2020; Li et al. 2021; Nguyen et al. 2021; Averyanov et al. 2022; Chen et al. 2022; Dey et al. 2022; Liao et al. 2022; Zhang et al. 2022, 2024a, b; Lee et al. 2023; Li et al. 2023; Liu et al. 2023; Ya et al. 2023; Zhou et al. 2024), confirmed that it represents a new species of *Gastrochilus*. In parallel, we investigated the phylogenetic position of *G.obovatifolius* sp. nov., which is here described and illustrated.

## ﻿Materials and methods

### ﻿Morphological analyses

Specimens of the novelty were collected during our field expedition to the Chongqing Municipality in 2024. Photographs were taken using a Nikon D7200 digital camera (Japan). Morphological descriptions and measurements were based on living plants and dried herbarium specimens deposited at CGMC and IBK (herbarium acronyms follow Thiers 2024). This material was compared to relevant specimens, including types, presented in Zhang et al. (2024b), housed at CDBI, K, KUN and PE. Descriptive terminology follows Beentje (2016).

### ﻿Genomic DNA extraction and sequencing

Leaf material for DNA extraction was dried using silica gel (Chase and Hills 1991). Genomic DNA was extracted using a modified CTAB protocol (Chen et al. 2014). The total genomic DNA sample was sent to Majorbio in China (http://www.majorbio.com/) for library construction and next-generation sequencing. Short-insert (350 bp) paired-end read libraries preparation and 2 × 150 bp sequencing were performed on an Illumina (HiSeq4000) genome analyzer platform. Approximately 1 Gb of raw data for the new species was filtered using the FASTX-Toolkit (http://hannonlab.cshl.edu/fastx_toolkit/download.html) to obtain high-quality clean data by removing adapters and low-quality reads.

### ﻿Plastid genome and ribosomal DNA (rDNA) assembly and annotation

Complete chloroplast genome and ribosomal genome data were assembled using GetOrganelle v.1.7.7.0 (Jin et al. 2020). ITS sequence extraction was performed by ITSx v.1.1.3 (Bengtsson-Palme et al. 2013). The plastid genome was preliminarily annotated using CPGAVAS2 (Shi et al. 2019), with *Gastrochilusfuscopunctatus* (Hayata) Hayata (GenBank: NC_035830) as the reference genome. The annotation results were confirmed using Geseq (Tillich et al. 2017). The genome map of the new species was drawn by OGDRAW (Greiner et al. 2019). The genome sequences were deposited in GenBank (accession numbers: PP942372, PP949380).

### ﻿Phylogenetic analyses

To investigate the phylogenetic position of this species, we extracted five DNA regions (ITS, *mat*K, *psb*A–*trn*H, *psb*M–*trn*D, and *trn*L–F) from assembled rDNA and complete plastid genome sequences of the new species. Additionally, we downloaded sequence data used in Zhang et al. (2024a) and Ya et al. (2023) from GenBank (Suppl. material 1). This resulted in 95 accessions representing 61 taxa in total, with 55 taxa belonging to *Gastrochilus* as the ingroup, and six Aeridinae species in the genera *Luisia* Gaudich., *Saccolabium* Blume, *Holcoglossum* Schltr. and *Pomatocalpa* Breda as the outgroup. All sequences were aligned using MAFFT and the five aligned DNA markers were concatenated in Phylosuite v.1.2.3 (Katoh and Standley 2013; Zhang et al. 2020; Xiang et al. 2023). The substitution model was determined in Phylosuite v.1.2.3 using ModelFinder (Kalyaanamoorthy et al. 2017; Zhang et al. 2020; Xiang et al. 2023) and the evolutionary best fit model (GTR+F+I+G4) was selected using the corrected Akaike Information Criterion (AICc). Bayesian Inference (BI) was conducted using MrBayes in Phylosuite v.1.2.3 (Ronquist et al. 2012; Zhang et al. 2020; Xiang et al. 2023). The Markov chains were run for 1,000,000 generations, with sampling every 1,000 generations and a burn-in of 0.25. Four Markov chains with two runs were executed. The Maximum Likelihood (ML) phylogenetic trees were generated in IQ-TREE with 1000 bootstrap replicates in Phylosuite v.1.2.3 (Guindon et al. 2010; Minh et al. 2013; Nguyen et al. 2015; Zhang et al. 2020; Xiang et al. 2023).

## ﻿Results

**Figure 1.**
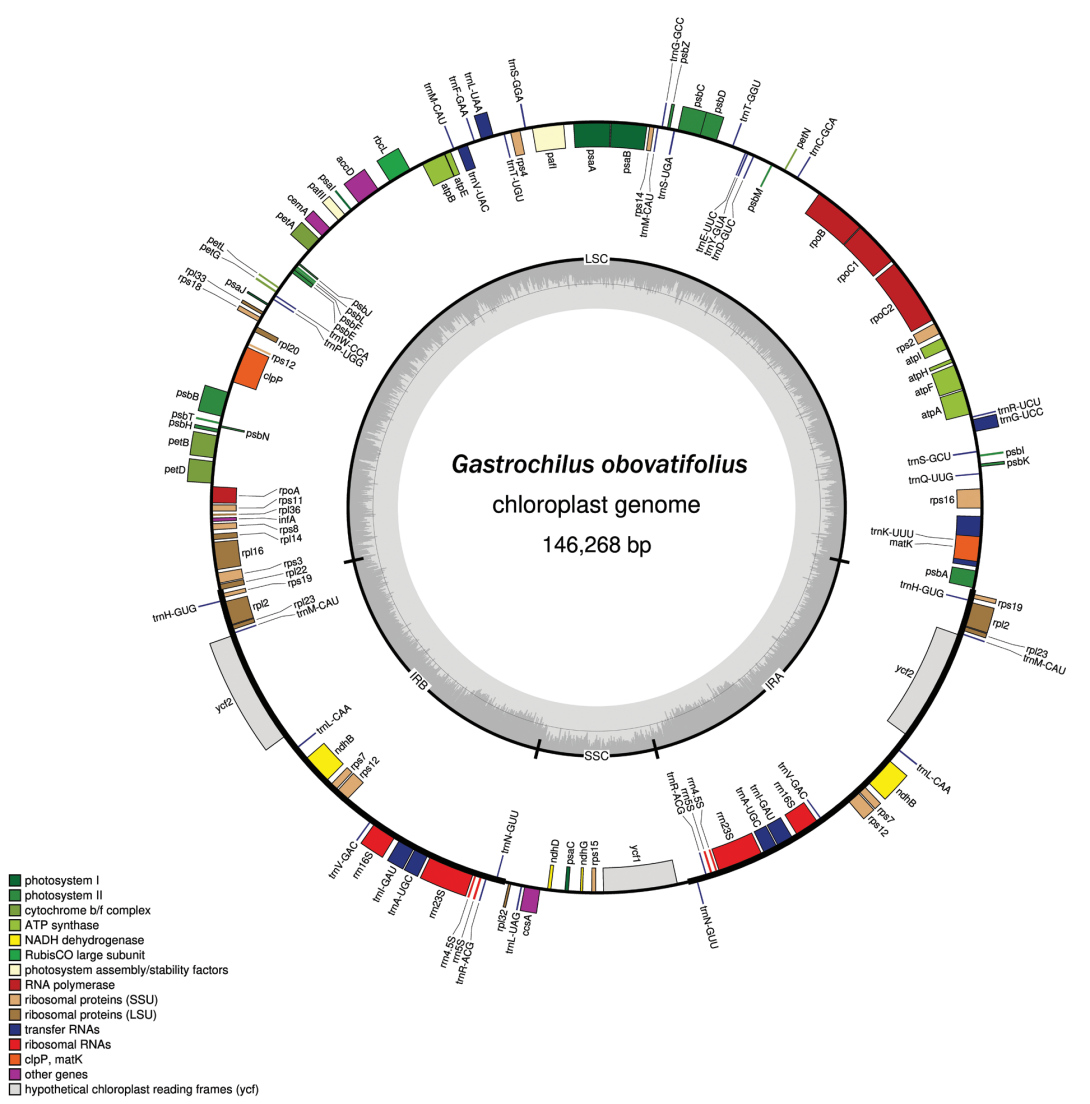
Plastome map of *Gastrochilusobovatifolius*. The thick lines on the outer complete circle represent the inverted repeat regions (IRa and IRb). The gray gradient in the innermost circle represents the GC content. Genes on the outside and inside of the map are transcribed in clockwise and counter directions, respectively.

**Figure 2.**
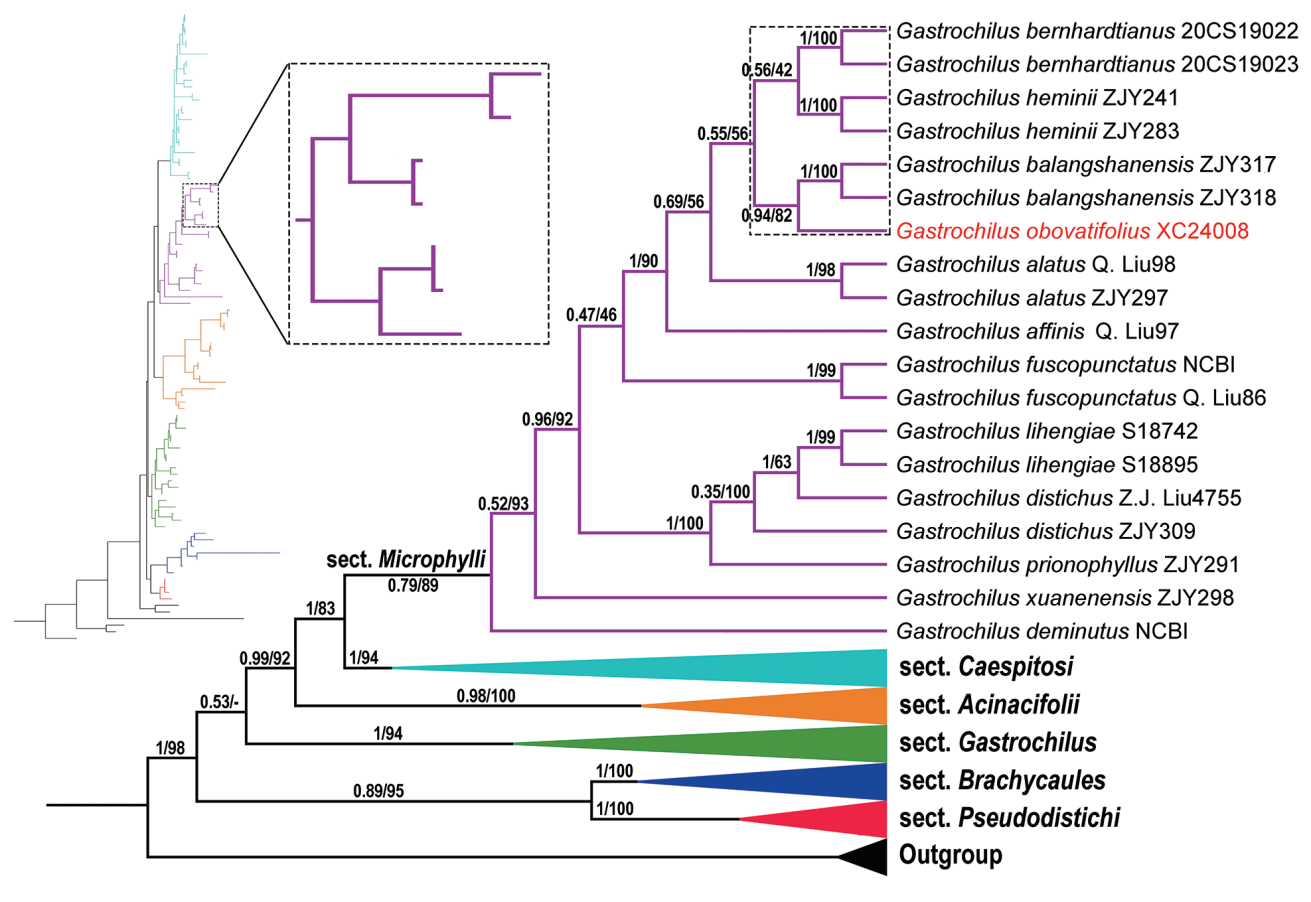
Bayesian tree from analysis of combined nrITS and four-plastid (*mat*K, *psb*A–*trn*H, *psb*M–*trn*D, and *trn*L–F) marker data of 55 species of *Gastrochilus*. The bootstrap values (BS) of ML and posterior probabilities (PP) of BI are listed at each node. The new species is highlighted in red, dotted lines shows its phylogenetic relationship with three related species.

The complete plastid genome sequences of *Gastrochilusobovatifolius* sp. nov. comprise 146,248 bp (Fig. 1). The characteristics and statistics of the plastid genome are summarized in Suppl. materials 2, 3. The aligned nrITS matrix is 683 nucleotides long with 207 variable sites, and the combined four plastid markers matrix including 3,431 nucleotides in length with 343 variable sites, consists of 805 bp for *mat*K, 677 bp for *psb*A–*trn*H, 950 bp for *psb*M–*trn*D, and 999 bp for *trn*L–F, respectively. The characteristics and statistics of the five plastid markers are summarized in Suppl. material 4. The resulting tree topology (Fig. 2) aligns with previous phylogenetic analyses of *Gastrochilus* (Zhang et al. 2024a, b). The new species and *G.balangshanensis* Jun Y.Zhang, B.Xu & Yue H.Cheng form a monophyletic pair (PP/BS = 0.94/82) sister to *G.heminii* and *G.bernhardtianus* (PP/BS = 0.56/42), within G.sect.Microphylli (PP/BS = 0.79/89) (Fig. 2).

A morphological comparison of *G.obovatifolius*, *G.balangshanensis*, *G.heminii*, and *G.affinis* is presented in Table 1, which further substantiates the recognition of *G.obovatifolius* as a new species.

**Table 1.** Morphological comparison between *Gastrochilusobovatifolius* and three morphologically similar species in G.sect.Microphylli.

Characters	*G.obovatifolius*	*G.affinis*	*G.balangshanensis*	*G.heminii*
Leaves	obovate, 1.4–1.6 × 0.6–0.8 cm	elliptic to narrowly oblong, 1.2–2.6 × 0.3–0.7 cm	nearly elliptic, 0.9–1.5 × 0.4–0.8 cm	narrowly oblong or oblong-falcate, 0.9–2.3 × 0.3–0.5 cm
Dorsal sepal	elliptic, ca. 5.0 × 4.0 mm, apex obtuse	elliptic-oblong, 3.0–5.0 × 1.0–1.3 mm, apex obtuse	elliptic, 5.6–6.4 × 4.8–5.2 mm, apex obtuse	elliptic-oblong, ca. 2.4 × 1.5 mm, apex obtuse
Lateral sepals	elliptic, slightly oblique, ca. 5.0 × 3.0 mm, apex obtuse	elliptic-ovate, slightly oblique and incurved, 3.5–4 × 0.7–1.3 mm, apex obtuse	elliptic, 5.6–6.4 × 4.8–5.2 mm, apex obtuse	elliptic-oblong, ca. 2.4 × 1.5 mm, apex obtuse
Petals	oblong, ca. 6.0 × 3.0 mm	ovate-elliptic to elliptic, 3.0.–4.0 × 1.0–1.3 mm	oblong, 5.0–5.8 × 4.0–4.4 mm	narrowly oblong, ca. 2.6 × 1.3 mm
Epichile	reniform, 10.0–12.0 × 6.0–8.0 mm, revolute, margin erose, median patch dark purple with 2 low ridges	subtriangular, ca. 8.0 × 4.5 mm, decurved, margin finely erose at base, median patch brown to purplish-brown, with 2 thick ridges	reniform, 10.0–12.0 × 5.5–6.5 mm, revolute, margin erose, median patch purple-red with two inconspicuous ridges	reniform, 4.2–6.5 × 2.0–3.0 mm, revolute, margin erose, median patch purple-red with irregular folds
Hypochile	sub-hemispherical, 4.0–4.5 × 4.0–4.2 mm, dorsally compressed, slightly bent outward, obtuse at the apex	obconical, 3.0–4.0 × 2.0–3.0 mm, dorsally compressed, slightly bent outward, subacute to obtuse and shortly bifid at apex	sub-hemispherical, 6.0–8.0 × 5.8–7.5 mm, dorsally compressed, obtuse-rounded at the apex	subconical, 2.0–2.4 × 1.6–2.0 mm, dorsally compressed, slightly bent outward, splits into two conical sacs at the apex
Distribution	Chengkou, NE. Chongqing	Gaoligongshan, NW. Yunnan, Xizang; India, Nepal	Wenchuan, Central Sichuan	Wenchuan, Central Sichuan

### ﻿Taxonomic treatment

#### ﻿ Gastrochilus obovatifolius

Taxon classificationPlantaeAsparagalesOrchidaceae

C.Xiong, X.Y.Fu & S.R.Yi sp.nov.

FEB62E9D-C5B9-557D-8C1A-BD9AFEDB2703

urn:lsid:ipni.org:names:77356371-1

Figs 3 , 4

##### Diagnosis.

*Gastrochilusobovatifolius* is most similar to *G.balangshanensis*., but differs by the longer stem (3–5 vs. 1.5–3.5 cm), obovate leaves (vs. nearly elliptic), and smaller sepals (ca. 5.0 × 3.0–4.0 vs. 5.6–6.4 × 4.8–5.2 mm) and hypochile (4.0–4.5 × 4.0–4.2 vs. 6.0–8.0 × 5.8–7.5 mm).

**Figure 3.**
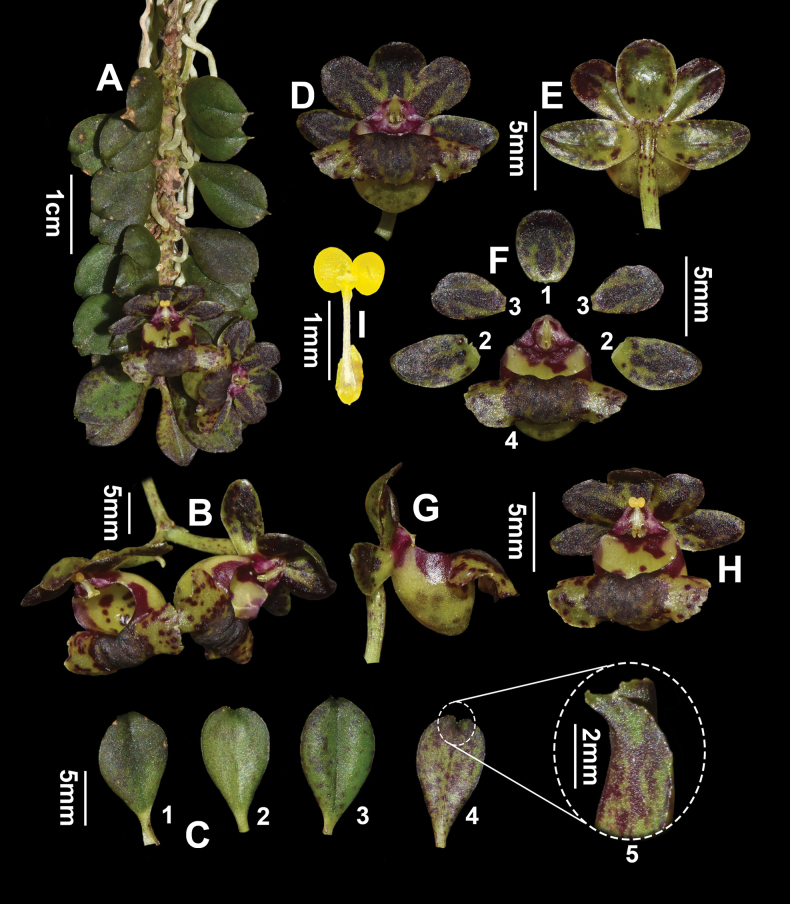
*Gastrochilusobovatifolius* C.Xiong, X.Y.Fu & S.R.Yi, sp. nov. *in vivo*. **A** flowering plant **B** inflorescence **C** leaves (C1–C3: front view, C4: back view, C5: close-up of leaf apex) **D** flower (front view) **E** flower (back view) **F** dissected flower (F1: dorsal sepal; F2: lateral sepals; F3: petals; F4: labellum) **G** flower (side view) **H** flower (top view) **I** pollinarium.

##### Type.

China • Chongqing: Chengkou County (城口县), Dong’an Town (东安镇), Lizishuping (栗子树坪), 31°42′N, 109°11′E, alt. ca. 1650 m, 30 March 2024, *Si-Rong Yi et al. YSR2703* (holotype IBK!, isotype CGMC!).

**Figure 4.**
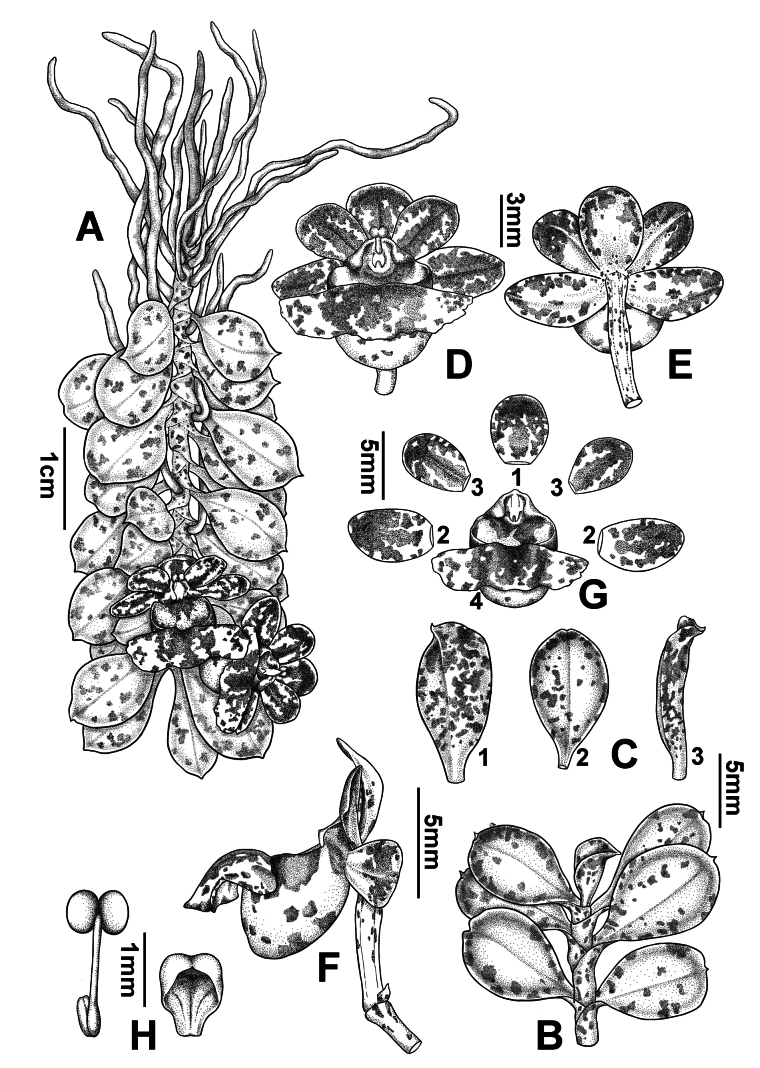
*Gastrochilusobovatifolius* C.Xiong, X.Y.Fu & S.R.Yi, sp. nov., an overview **A** habit **B** stem apex **C** leaves (C1: front view, C2: back view, C3: side view) **D** flower (side view) **E** flower (front view) **F** flower (back view) **G** dissected flower (G1: dorsal sepal; G2: lateral sepals; G3: petals; G4: labellum) **H** pollinarium and anther cap.

##### Description.

***Epiphytic herbs***, monopodial, pendent, with a short stem, 3.0–5.0 cm long, leafy. ***Roots*** vermiform, slender, 4.0–6.0 cm long and ca. 1 mm in diameter. ***Stems*** short, branched, green, glabrous, up to 5 cm long and ca. 2.0 mm in diameter, covered with sheathing leaf bases; sheaths with purplish-red spots; internodes 1.2–1.8 mm long. ***Leaves*** alternate, distichous, obovate, fleshy, 1.4–1.6 × 0.6–0.8 cm, margin entire, apex acute, obscurely serrate; young leaves yellow-green with a few purplish-red spots, mature leaves green with hardly any purplish-red spots. ***Inflorescences*** 1–2 racemes, usually arising from the axil of an upper leaf, 2.0–2.5 cm long, 1–2-flowered; peduncle 0.8–1.2 cm long; bracts 1.0–2.0 mm long. ***Flowers*** 1.2–1.4 × 1.0–1.2 cm, yellow-green, with dark purple stripes on petals and sepals, raised abaxially along the midrib; pedicel and ovary yellowish-green with purple-red spots, 1.5–1.6 cm long. ***Dorsal sepal*** elliptic, concave, ca. 5.0 × 4.0 mm, apex obtuse; ***lateral sepals*** similar to dorsal sepal, slightly oblique, ca. 5.0 × 3.0 mm, apex obtuse; petals oblong, concave, ca. 6.0 × 3.0 mm, apex obtuse. ***Labellum*** epichile reniform, yellow-green with purplish-red spots, 1.0–1.2 × 0.6–0.8 cm, revolute, margin erose, smooth and glabrous above, median patch, thickened, with 2 low ridges, dark purple; hypochile sub-hemispherical, yellow-green, mouth with lateral purplish-red markings, sac with purplish-red spots on the underside, obtuse at the apex, 4.0–4.5 mm tall, 4.0–4.2 mm in diameter, dorsally compressed, slightly bent outward. ***Column*** stout, ca. 2.0 × 1.2 mm; rostellum bilobed; anther cap galeate with curved emarginate beak, ca. 1.0 × 1.2 mm; pollinia 2, ca. 0.8 × 0.6 mm, yellow, nearly spherical, entire, with a depression in the center; stipe elongate, ca. 1.2 mm long, viscidium yellow, elliptic, ca. 0.8 × 0.4 mm. ***Capsule*** not seen.

##### Phenology.

Flowering from March to April.

##### Etymology.

The specific epithet ‘*obovatifolius*’ refers to the highly distinctive obovate leaves. The suggested Chinese common name is “dào luǎn yè pén jù lán (倒卵叶盆距兰)”.

##### Distribution and ecology.

**Figure 5.**
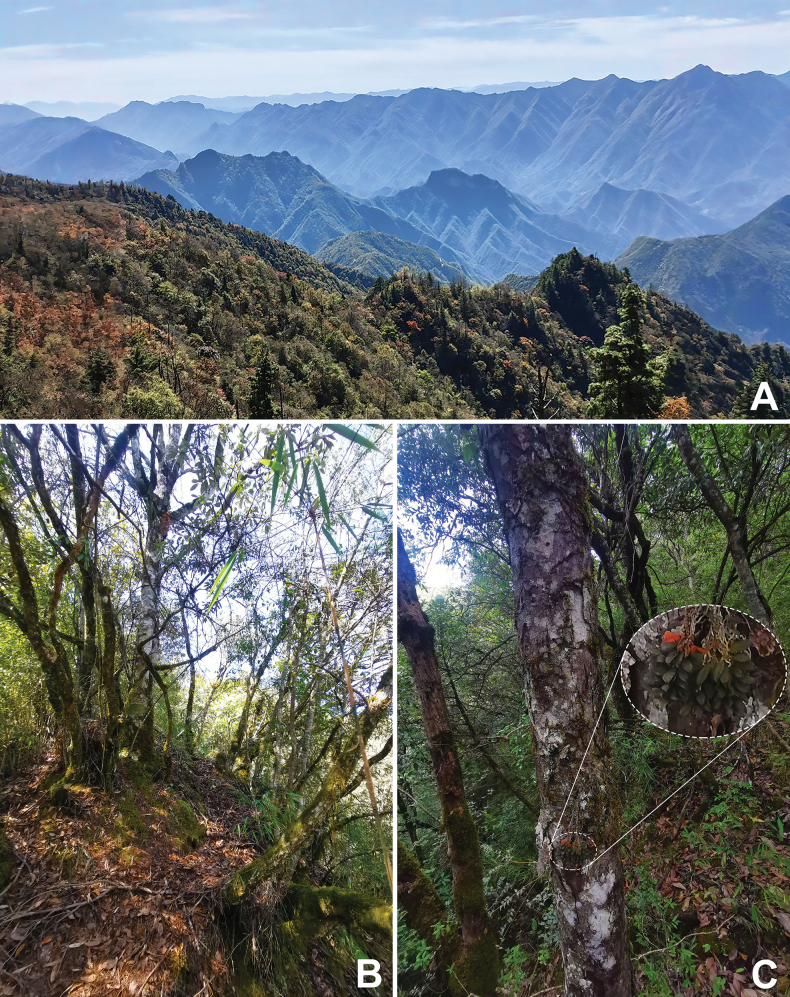
*Gastrochilusobovatifolius*, habitat and plants in situ. **A** broad-leaved temperate forest in the Daba Mountains of Chongqing **B** a woodland trail lined with *Fargesiaspathacea*, *Quercusengleriana*, *Pierisjaponica*, *Rhododendronadenopodum*, and *Zanthoxylumdimorphophyllum***C***G.obovatifolius* growing as a trunk epiphyte on *Q.engleriana*.

**Figure 6.**
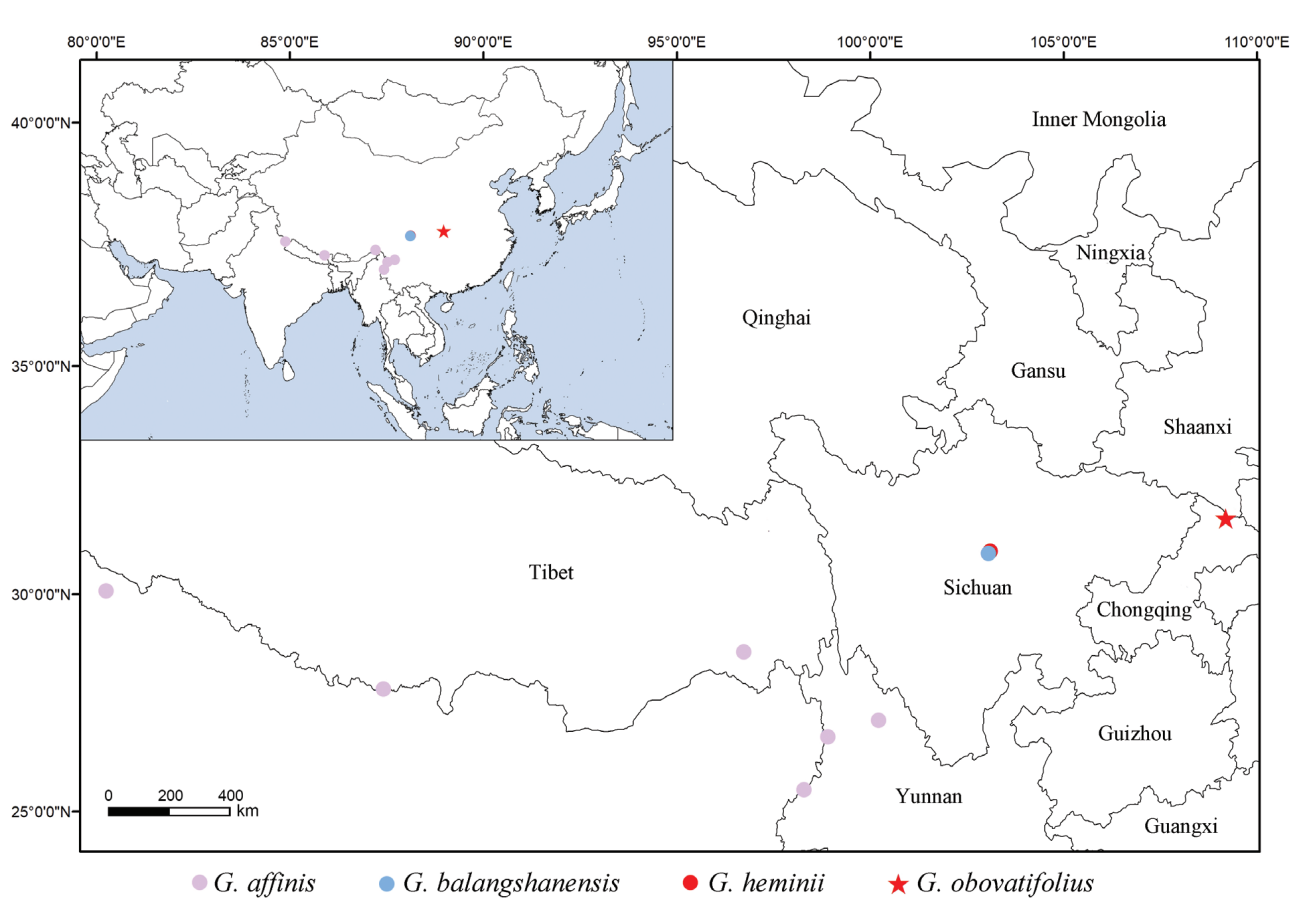
Distribution map of *Gastrochilusobovatifolius* and three related species of G.sect.Microphylli in southwestern China and adjoining areas.

The new species has only been recorded in Chengkou County, northeast Chongqing Municipality, bordering Shaanxi Province, China (Fig. 6). It grows as a trunk epiphyte on *Quercusengleriana* Seemen, in evergreen broad-leaved forest at an elevation of 1620–1650 m a.s.l. (Fig. 5). Besides *Q.engleriana*, the most frequent co-occurring angiosperm species include *Fargesiaspathacea* Franch., *Hepaticahenryi* (Oliv.) Steward, *Pierisjaponica* (Thunb.) D. Don ex G. Don, *Rhododendronadenopodum* Franch., and *Zanthoxylumdimorphophyllum* Hemsl.

##### Additional specimens examined (paratypes).

China • Chongqing: Chengkou County (城口县), Dong’an Town (东安镇), Dongjiachang (董家厂), 31°42′N, 109°12′E, alt. ca. 1620 m, 8 April 2024, *Si-Rong Yi et al. YSR2733* (IBK!, CGMC!).

## ﻿Discussion

The phylogenetic analysis revealed that the newly discovered *Gastrochilusobovatifolius*, along with 12 other *Gastrochilus* species (comprising 19 accessions), form a clade classified as Gastrochilussect.Microphylli. This clade is distinguished by floral characteristics such as diminutive flowers (petals and sepals < 6 mm long), a nearly reniform glabrous epichile with central irregular projections, and in some species, a hypochile split into two conical protrusions (Zhang et al. 2024a). The obovate leaves of *G.obovatifolius* set it apart from any other species in Gastrochilussect.Microphylli, which predominantly exhibit lanceolate, oblong, or ovate leaves.

Gastrochilussect.Microphylli now encompasses a total of 16 species, including 13 distributed in southern and southwestern China. Among these 13 species, only *G.xuanenensis* Z. H. Tsi (Tsi 1982) and the newly described *G.obovatifolius* are known to be restricted to the Qinling-Daba Mountains (QDM), while the Hengduan Mountains (HDM) harbor a greater diversity, with 11 out of the 13 identified species (Zhang et al. 2024b). The high endemism rate in the QDM and its adjacent regions (Zhang et al. 2022a, b), contrasts with the relatively low diversity of G.sect.Microphylli species in this area, which may suggest some potential for the identification of new species. Accordingly, there is a pressing need for further field research and investigations focused on *Gastrochilus* and other epiphytic orchids in this region.

In Chengkou County, where *G.obovatifolius* was discovered, two other species of *Gastrochilus* are present: *G.fargesii* (Kraenzl.) Schltr. and *G.formosanus* (Hayata) Hayata. These species are classified under G.sect.Acinacifolii and G.sect.Caespitosi, respectively. They can be easily differentiated from *G.obovatifolius* by their foliar and floral characteristics, including the longer leaves (5–15 cm long) and the differently textured epichile, which is densely covered with papillate hairs.

## Supplementary Material

XML Treatment for Gastrochilus obovatifolius

## ﻿Additional information

### Author ORCIDs

Chi Xiong https://orcid.org/0000-0002-9082-2539

Xiao-Ying Fu https://orcid.org/0009-0009-1090-877X

Ke Tan https://orcid.org/0000-0002-9036-163X

Ya Huang https://orcid.org/0000-0003-3939-4046

Hong-Jing Zhang https://orcid.org/0000-0003-0025-9269

Yi-Chen Wang https://orcid.org/0000-0001-9541-975X

Yu-Bing Yang https://orcid.org/0000-0002-2742-6701

Si-Rong Yi https://orcid.org/0000-0002-3883-6175

### Data availability

All of the data that support the findings of this study are available in the main text or Supplementary Information.

## Supplementary materials

Supplementary material 1 Detailed information of material, voucher and GenBank accession numbers Data type docx https://binary.pensoft.net/file/1244763This dataset is made available under the Open Database License (http://opendatacommons.org/licenses/odbl/1.0/). The Open Database License (ODbL) is a license agreement intended to allow users to freely share, modify, and use this Dataset while maintaining this same freedom for others, provided that the original source and author(s) are credited.Chi Xiong, Xiao-Ying Fu, Ke Tan, Ya Huang, Hong-Jing Zhang, Yi-Chen Wang, Yu-Bing Yang, Si-Rong Yi

Supplementary material 2 Genes encoded in the plastid genome of *Gastrochilusobovatifolius* C.Xiong, X.Y.Fu & S.R.Yi Data type docx https://binary.pensoft.net/file/1244764This dataset is made available under the Open Database License (http://opendatacommons.org/licenses/odbl/1.0/). The Open Database License (ODbL) is a license agreement intended to allow users to freely share, modify, and use this Dataset while maintaining this same freedom for others, provided that the original source and author(s) are credited.Chi Xiong, Xiao-Ying Fu, Ke Tan, Ya Huang, Hong-Jing Zhang, Yi-Chen Wang, Yu-Bing Yang, Si-Rong Yi

Supplementary material 3 Summary of the whole plastid genome of *Gastrochilusobovatifolius* C.Xiong, X.Y.Fu & S.R.Yi Data type docx https://binary.pensoft.net/file/1244765This dataset is made available under the Open Database License (http://opendatacommons.org/licenses/odbl/1.0/). The Open Database License (ODbL) is a license agreement intended to allow users to freely share, modify, and use this Dataset while maintaining this same freedom for others, provided that the original source and author(s) are credited.Chi Xiong, Xiao-Ying Fu, Ke Tan, Ya Huang, Hong-Jing Zhang, Yi-Chen Wang, Yu-Bing Yang, Si-Rong Yi

Supplementary material 4 Statistics for the molecular datasets used in this study Data type docx https://binary.pensoft.net/file/1244766This dataset is made available under the Open Database License (http://opendatacommons.org/licenses/odbl/1.0/). The Open Database License (ODbL) is a license agreement intended to allow users to freely share, modify, and use this Dataset while maintaining this same freedom for others, provided that the original source and author(s) are credited.Chi Xiong, Xiao-Ying Fu, Ke Tan, Ya Huang, Hong-Jing Zhang, Yi-Chen Wang, Yu-Bing Yang, Si-Rong Yi
