# Supplementary material for: ﻿Gastrochilusobovatifolius (Orchidaceae, Aeridinae), a new species from the Daba Mountains of Chongqing, China
Source: PhytoKeys. 2025 Feb 7;252:25–40. doi: 10.3897/phytokeys.252.133501 (PMC11829196; doi:10.3897/phytokeys.252.133501)
Supplement: Supplementary material 1 — Detailed information of material, voucher and GenBank accession numbers [file phytokeys-252-025_article-133501__-s001.docx]

**Table S1.** Detailed information of material, voucher and GenBank accession numbers.

| Taxa | Voucher | nrITS | *mat*K | *psb*A–*trn*H | *trn*L–F | *psb*M–*trn*D |
| --- | --- | --- | --- | --- | --- | --- |
| *Gastrochilus acaulis* | — | KM583455 | KM583465 | — | — | — |
| *Gastrochilus acinacifolius* | Z. J. Liu 3316 | KJ733412 | KJ733569 | KJ733492 | KJ733649 | — |
| *Gastrochilus acinacifolius* | Q. Liu 62 | MK357118 | MK357138 | MK357160 | MK357208 | MK357216 |
| *Gastrochilus acinacifolius* | ZJY289 | OQ566796 | OQ575562 | OQ575590 | OQ575530 | OQ575619 |
| *Gastrochilus acutifolius* | Q. Liu 05 | — | MK357140 | MK357162 | — | MK357230 |
| *Gastrochilus acutifolius* | JK-DEBCR-mat-31(JK) | MW475270 | MW433889 | — | — | — |
| *Gastrochilus affinis* | Q. Liu 97 | — | MK357141 | MK357163 | — | MK357227 |
| *Gastrochilus alatus* | Q. Liu 98 | — | — | — | — | MK357228 |
| *Gastrochilus alatus* | ZJY297 | — | OQ575563 | OQ575591 | OQ575531 | OQ575620 |
| *Gastrochilus armeniacus* | ZJY244 | OP348889 | OP373113 | OP373119 | OP373128 | OP373123 |
| *Gastrochilus armeniacus* | ZJY285 | OP348888 | OP373114 | OP373118 | OP373127 | OP373124 |
| *Gastrochilus balangshanensis* | ZJY317 | PP960151 | PP944493 | PP944495 | PP944499 | PP944497 |
| *Gastrochilus balangshanensis* | ZJY318 | PP960152 | PP944494 | PP944496 | PP944500 | PP944498 |
| *Gastrochilus bellinus* | Q. Liu 52 | KY966597 | KY966884 | — | — | — |
| *Gastrochilus bellinus* | Q. Liu 53 | MK357123 | MK357142 | MK357164 | MK357202 | MK357241 |
| *Gastrochilus bellinus* | ZJY279 | OQ566799 | OQ575564 | OQ575592 | OQ575534 | OQ575621 |
| *Gastrochilus bernhardtianus* | 20CS19023 | OR073405 | OR772951 | OR772951 | OR772951 | OR772951 |
| *Gastrochilus bernhardtianus* | 20CS19022 | OR073404 | OR002167 | OR002167 | OR002167 | OR002167 |
| *Gastrochilus bigibbus* | Li 009 | — | MN124439 | MN124439 | MN124439 | MN124439 |
| *Gastrochilus calceolaris* | Z. J. Liu 3769 | KF545874 | KF545885 | KF545865 | KF545896 | — |
| *Gastrochilus calceolaris* | Q. Liu 94 | MK357126 | MK357144 | MK357169 | MK357205 | MK357233 |
| *Gastrochilus calceolaris* | ZJY281 | — | OQ575565 | OQ575593 | OQ575535 | OQ575622 |
| *Gastrochilus changjiangensis* | Q. Liu 45 | MK357124 | — | MK357166 | — | MK357236 |
| *Gastrochilus ciliaris* | Q. Liu 88 | — | MK357148 | MK357173 | — | MK357225 |
| *Gastrochilus ciliaris* | ZJY299 | — | OQ575566 | OQ575594 | — | OQ575623 |
| *Gastrochilus dasypogon* | Q. Liu 30 | MK357129 | MK357149 | MK357181 | MK357197 | MK357219 |
| *Gastrochilus deminutus* | — | KY966600 | KY966887 | — | — | — |
| *Gastrochilus distichus* | Z. J. Liu 4755 | KJ733414 | KJ733571 | KJ733494 | KJ733651 | — |
| *Gastrochilus distichus* | ZJY309 | OQ566800 | OQ575567 | OQ575595 | OQ575536 | — |
| *Gastrochilus fargesii* | 20HT3548 | — | OR002175 | OR002175 | OR002175 | OR002175 |
| *Gastrochilus formosanus* | Z. J. Liu 4265 | KJ733416 | KJ733573 | KJ733495 | KJ733653 | — |
| *Gastrochilus formosanus* | Q. Liu 91 | — | — | MK357174 | — | MK357226 |
| *Gastrochilus formosanus* | ZJY276 | OQ566801 | OQ575568 | OQ575596 | OQ575537 | OQ575624 |
| *Gastrochilus fuscopunctatus* | Q. Liu 86 | — | MK357150 | MK357171 | MK357192 | MK357231 |
| *Gastrochilus fuscopunctatus* | — | — | KX871233 | KX871233 | KX871233 | KX871233 |
| *Gastrochilus gongshanensis* | ZJY295 | — | OQ575569 | OQ575597 | OQ575538 | OQ575625 |
| *Gastrochilus guangtungensis* | Z. J. Liu 4127 | KJ733417 | KJ733574 | KJ733496 | KJ733654 | — |
| *Gastrochilus guangtungensis* | ZJY293 | OQ566802 | OQ575570 | OQ575598 | OQ575539 | OQ575626 |
| *Gastrochilus heminii* | ZJY241 | ON286752 | ON331126 | ON331128 | ON331132 | ON331130 |
| *Gastrochilus heminii* | ZJY283 | ON286753 | ON331127 | ON331129 | ON331133 | ON331131 |
| *Gastrochilus intermedius* | Q. Liu 38 | MK357121 | MK357151 | MK357172 | MK357190 | MK357213 |
| *Gastrochilus japonicus* | Q. Liu 87 | KF545875 | KF545886 | KF545866 | KF545897 | — |
| *Gastrochilus japonicus* | — | — | KX871236 | KX871236 | KX871236 | KX871236 |
| *Gastrochilus kadooriei* | ZJY268 | OQ566803 | OQ575571 | OQ575599 | OQ575540 | OQ575627 |
| *Gastrochilus lihengiae* | 22CS21828 | OR073408 | OR002168 | OR002168 | OR002168 | OR002168 |
| *Gastrochilus lihengiae* | 23CS24145 | — | OR002169 | OR002169 | OR002169 | OR002169 |
| *Gastrochilus linearifolius* | Q. Liu 711 | MK357133 | MK357136 | MK357187 | MK357194 | MK357229 |
| *Gastrochilus linii* | Q. Liu 92 | — | MK357152 | MK357176 | MK357198 | MK357224 |
| *Gastrochilus malipoensis* | Q. Liu 71 | — | MK357147 | MK357177 | MK357200 | MK357235 |
| *Gastrochilus malipoensis* | ZJY288 | OQ566804 | OQ575572 | OQ575600 | OQ575541 | OQ575628 |
| *Gastrochilus matsuran* | — | KT338700 | — | — | — | — |
| *Gastrochilus minjiangensis* | ZJY243 | OP348887 | OP373112 | OP373117 | OP373126 | OP373122 |
| *Gastrochilus minjiangensis* | ZJY284 | OQ566806 | — | — | OQ575542 | — |
| *Gastrochilus minutiflorus* | Q. Liu 37 | — | MK357153 | MK357179 | — | MK357215 |
| *Gastrochilus minutiflorus* | ZJY287 | OQ566807 | OQ575573 | OQ575601 | OQ575544 | OQ575629 |
| *Gastrochilus nanchuanensis* | ZJY296 | OQ566808 | OQ575574 | OQ575602 | OQ575545 | OQ575630 |
| *Gastrochilus obliquus* var. *obliquus* | Q. Liu 708 | MK357131 | MK357137 | KJ733498 | KJ733656 | MK357211 |
| *Gastrochilus obliquus* var. *obliquus* | Q. Liu 44 | MK357130 | MK357154 | MK357182 | MK357195 | MK357218 |
| *Gastrochilus obliquus* var. *suavis* | ZJY277 | OQ566809 | OQ575575 | OQ575603 | OQ575546 | OQ575631 |
| *Gastrochilus obliquus* var. *suavis* | ZJY282 | OQ566810 | OQ575576 | OQ575604 | OQ575547 | OQ575632 |
| *Gastrochilus obovatifolius* | XC24008 | PP949380 | PP942372 | PP942372 | PP942372 | PP942372 |
| *Gastrochilus platycalcaratus* | Q. Liu 19 | MK357122 | — | MK357175 | — | MK357222 |
| *Gastrochilus platycalcaratus* | ZJY300 | OQ566811 | — | OQ575605 | OQ575548 | OQ575633 |
| *Gastrochilus prionophyllus* | ZJY291 | OQ566812 | OQ575577 | OQ575606 | OQ575549 | OQ575634 |
| *Gastrochilus pseudodistichus* | ZJY308 | OQ566813 | OQ575578 | OQ575607 | OQ575550 | OQ575635 |
| *Gastrochilus pseudodistichus* | ZJY290 | OQ566814 | OQ575579 | OQ575608 | OQ575551 | OQ575636 |
| *Gastrochilus rantabunensis* | Q. Liu 89 | — | MK357155 | MK357184 | MK357193 | MK357223 |
| *Gastrochilus rantabunensis* | ZJY303 | OQ566815 | OQ575580 | OQ575609 | OQ575552 | OQ575637 |
| *Gastrochilus raraensis* | Z. J. Liu 4798 | KJ733420 | KJ733577 | KJ733499 | KJ733657 | — |
| *Gastrochilus setosus* | ZJY304 | OQ566816 | OQ575581 | OQ575610 | OQ575553 | — |
| *Gastrochilus sinensis* | ZJY228 | OM985813 | OK042953 | OK172399 | OK172401 | OQ575646 |
| *Gastrochilus sinensis* | S1109 | OP348890 | OP373115 | OP373120 | OP373129 | OP373125 |
| *Gastrochilus somai* | Q. Liu 36 | MK357128 | — | MK357180 | — | MK357220 |
| *Gastrochilus somai* | Li 073 | — | MN124436 | MN124436 | MN124436 | MN124436 |
| *Gastrochilus sp.* | ZJY274 | OQ566817 | OQ575582 | OQ575611 | OQ575554 | OQ575638 |
| *Gastrochilus sumartranus* | ZJY280 | OQ566818 | OQ575583 | OQ575612 | OQ575555 | OQ575639 |
| *Gastrochilus sumartranus* | ZJY294 | OQ566819 | OQ575584 | OQ575613 | OQ575556 | OQ575640 |
| *Gastrochilus tianbaoensis* | ZJY275 | — | OQ575585 | OQ575614 | OQ575557 | OQ575641 |
| *Gastrochilus tianbaoensis* | Q. Liu 63 | MK357120 | MK357157 | MK357186 | MK357207 | MK357214 |
| *Gastrochilus tsii* | Li 076 | — | MN124437 | MN124437 | MN124437 | MN124437 |
| *Gastrochilus wenshanensis* | ZJY292 | OQ566820 | OQ575586 | OQ575615 | OQ575558 | OQ575642 |
| *Gastrochilus wolongensis* | ZJY240 | OM985810 | OK172400 | OK172402 | OK172404 | OK172403 |
| *Gastrochilus wolongensis* | S1108 | OM985811 | OM974209 | OM974211 | OM974210 | OQ575647 |
| *Gastrochilus xuanenensis* | ZJY298 | — | OQ575587 | OQ575616 | OQ575559 | OQ575643 |
| *Gastrochilus xizangensis* | — | OP740686 | — | — | — | — |
| *Gastrochilus yunlongensis* | ZJY301 | — | OQ575588 | OQ575617 | OQ575560 | OQ575644 |
| *Gastrochilus yunnanensis* | Q. Liu 60 | MK165469 | MK357158 | MK357185 | — | MK357212 |
| *Gastrochilus yunnanensis* | ZJY278 | OQ566821 | OQ575589 | OQ575618 | OQ575561 | OQ575645 |
| *Gastrochilus zhenyuanensis* | Q. Liu 61 | MK357127 | MK357146 | MK357168 | MK357199 | MK357237 |
| *Holcoglossum amesianum* | X.H. Jin 004/006 | HQ404389 | JF763779 | HQ404439 | — | — |
| *Holcoglossum kimballianum* | X.H. Jin 017 | HQ452901 | JF763787 | HQ404452 | — | — |
| *Luisia magniflora* | Z. J. Liu 3444 | KJ733426 | KJ733583 | KJ733505 | KJ733663 | — |
| *Pomatocalpa diffusum* | TBG145837 | AB217576 | AB217752 | — | EF670432 | — |
| *Pomatocalpa spicatum* | Z. J. Liu 4589 | KJ733438 | KJ733595 | KJ733518 | KJ733675 | — |
| *Saccolabium pusillum* | TBG144220 | AB217580 | AB217756 | — | — | — |
