# Supplementary material for: ﻿Gastrochilusobovatifolius (Orchidaceae, Aeridinae), a new species from the Daba Mountains of Chongqing, China
Source: PhytoKeys. 2025 Feb 7;252:25–40. doi: 10.3897/phytokeys.252.133501 (PMC11829196; doi:10.3897/phytokeys.252.133501)
Supplement: Supplementary material 2 — Genes encoded in the plastid genome of Gastrochilusobovatifolius C.Xiong, X.Y.Fu & S.R.Yi [file phytokeys-252-025_article-133501__-s002.docx]

**Table S2.** Genes encoded in the plastid genome of *Gastrochilus obovatifolius* C.Xiong, X.Y.Fu & S.R.Yi.

| Category | Group of genes | Gene name |
| --- | --- | --- |
| Photosynthesis | Subunits of photosystem I | *psa*A, *psa*B, *psa*C, *psa*I, *psa*J |
|  | Subunits of photosystem II | *psb*A, *psb*B, *psb*C, *psb*D, *psb*E, *psb*F, *psb*H, *psb*I, *psb*J, *psb*K, *psb*L, *psb*M, *psbN*, *psb*T, *psb*Z |
|  | Subunits of NADH dehydrogenase | *ndh*B*(×2), *ndh*D, *ndh*G |
|  | Subunits of cytochrome b/f complex | *pet*A, *pet*B*, *pet*D*, *pet*G, *pet*L, *pet*N |
|  | Subunits of ATP synthase | *atp*A, *atp*B, *atp*E, *atp*F*, *atp*H, *atp*I |
|  | Large subunit of rubisco | *rbc*L |
| Self-replication | Proteins of large ribosomal subunit | *rpl*2*(×2), *rpl*14, *rpl*16*, *rpl*20, *rpl*22, *rpl*23(×2), *rpl*32*, rpl*33*, rpl*36 |
|  | Proteins of small ribosomal subunit | *rps*2, *rps*3, *rps*4, *rps*7(×2), *rps*8, *rps*11, *rps*12**(×2), *rps*14, *rps*15, *rps*16*, *rps*18, *rps*19(×2) |
|  | Subunits of RNA polymerase | *rpo*A, *rpo*B, *rpo*C1*, *rpo*C2 |
|  | Ribosomal RNAs | *rrn*4.5(×2), *rrn*5(×2), *rrn*16(×2), *rrn*23(×2) |
|  | Transfer RNAs | *trn*A-UGC*(×2), *trn*C-GCA, *trn*D-GUC, *trn*E-UUC, *trn*F-GAA, *trn*G-GCC*, *trn*G-UCC, *trn*H-GUG(×2), *trn*I-GAU*(×2), *trn*K-UUU*, *trn*L-CAA(×2), *trn*L-UAA*, *trn*L-UAG, *trn*M-CAU(×2), *trn*N-GUU(×2), *trn*P-UGG, *trn*Q-UUG, *trn*R-ACG(×2), *trn*R-UCU, *trn*S-GCU, *trn*S-GGA, *trn*S-UGA, *trn*T-GGU, *trn*T-UGU, *trn*V-GAC(×2), *trn*V-UAC*, *trn*W-CCA, *trn*Y-GUA |
| Other genes | Maturase | *mat*K |
|  | Protease | *clp*P** |
|  | Envelope membrane protein | *cem*A |
|  | Acetyl-CoA carboxylase | *acc*D |
|  | c-type cytochrome synthesis gene | *ccs*A |
|  | Translation initiation factor | *inf*A |
| Genes of unknown function | Conserved hypothetical chloroplast ORF | *ycf*1, *ycf*2(×2) |
| Notes: Gene*: Gene with one introns; Gene**: Gene with two introns; Gene(×2): Number of copies of multi-copy genes. | | |
