# Supplementary material for: ﻿Gastrochilusobovatifolius (Orchidaceae, Aeridinae), a new species from the Daba Mountains of Chongqing, China
Source: PhytoKeys. 2025 Feb 7;252:25–40. doi: 10.3897/phytokeys.252.133501 (PMC11829196; doi:10.3897/phytokeys.252.133501)
Supplement: Supplementary material 3 — Summary of the whole plastid genome of Gastrochilusobovatifolius C.Xiong, X.Y.Fu & S.R.Yi [file phytokeys-252-025_article-133501__-s003.docx]

**Table S3.** Summary of the whole plastid genome of *Gastrochilus obovatifolius* C.Xiong, X.Y.Fu & S.R.Yi.

| Characteristic | *Gastrochilus obovatifolius* |
| --- | --- |
| size(bp) | 146,268 |
| LSC length (bp) | 83,135 |
| SSC length (bp) | 11,147 |
| IR length (bp) | 25,993 |
| Number of genes | 101 |
| Protein-coding genes | 69 |
| rRNA genes | 4 |
| tRNA genes | 28 |
| LSC GC% | 34.02% |
| SSC GC% | 28.11% |
| IR GC% | 43.06% |
