# Supplementary material for: ﻿Gastrochilusobovatifolius (Orchidaceae, Aeridinae), a new species from the Daba Mountains of Chongqing, China
Source: PhytoKeys. 2025 Feb 7;252:25–40. doi: 10.3897/phytokeys.252.133501 (PMC11829196; doi:10.3897/phytokeys.252.133501)
Supplement: Supplementary material 4 — Statistics for the molecular datasets used in this study [file phytokeys-252-025_article-133501__-s004.docx]

**Table S4.** Statistics for the molecular datasets used in this study.

|  | Number of  sequences | Aligned length (bp) | Variable characters (bp) | Parsimony information characters (bp) |
| --- | --- | --- | --- | --- |
| ITS | 71 | 683 | 207 | 135 |
| *mat*K | 86 | 805 | 113 | 53 |
| *psb*A-*trn*H | 85 | 677 | 30 | 19 |
| *psb*M-*trn*D | 73 | 950 | 82 | 62 |
| *trn*L-F | 75 | 999 | 118 | 57 |
| Combined | 95 | 4,114 | 550 | 326 |
